# Recurrent climate related extreme events and patterns of race/ethnic mortality: opportunities to advance population health equity^☆^^☆☆^

**DOI:** 10.1016/j.joclim.2026.100674

**Published:** 2026-04-15

**Authors:** Dorothy M. Daley, Margaret H. Swenson, Jarron M. Saint Onge

**Affiliations:** a University of Kansas, School of Public Affairs and Administration and the Environmental Studies Program, 1445 Jayhawk Blvd. Lawrence, KS 66045 USA; b University of Wyoming, School of Politics, Public Affairs & International Studies, 1000 E. University Ave. Laramie, WY 82071, USA; c University of Kansas, Department of Sociology/ Population Health, 1415 Jayhawk Blvd. Lawrence KS, 66045 USA

**Keywords:** Climate change, Population health, Health equity, Social determinants of health, Premature Mortality, Counties

## Abstract

**Introduction:**

Climate change increases the frequency and intensity of droughts, excessive heat, damaging storms and other extreme events. In the United States, economic and racial inequality, combined with widespread divestment in communities, exacerbate the health risks from climate change through recurrent and overlapping climate extremes. Yet, the population health consequences of recurrent climate risks are not well understood.

**Methods:**

Using existing environmental and climate data from several sources, we develop a novel measure of recurrent climate extreme events from 2010–2019. We merge this with population health data from the County Health Rankings and Roadmap project. Our analysis examines the association between recurrent climate extreme events and age-adjusted premature mortality across two subgroups, non-Hispanic Black and non-Hispanic White residents.

**Results:**

Controlling for established determinants of population health, climate extremes are associated with higher levels of premature mortality for non-Hispanic Black residents, but within the same counties, climate extremes are not consistently related to White premature mortality. Specifically, results showed a statistically significant 37% increase in premature mortality for multiple decadal climate events for non-Hispanic Black populations.

**Conclusions:**

Local communities experience and respond to climate-related events, but within the same locality, climate extremes can magnify health disparities. Policies that integrate climate change and health must be informed by local data that help identify and support populations within a community made differentially vulnerable to climate change.

## 1 Introduction

Climate change remains a profound environmental and population health challenge. As climate change continues, the intensity and frequency of droughts, floods, heat waves and other extreme events will likely increase[[1], [2], [3]], along with climate-related risks of physical and mental illnesses, exposures to diseases, and subsequent deaths. The health implications of climate change are complex, and they are likely to operate via multiple pathways, over varying time scales, and have differential impacts on disease. Low-income communities, communities of color, and Indigenous communities are at particular risk for deleterious effects of climate change[[4], [5], [6]], in part due to limited material resources and systematic erosion of protective resources and public services to mitigate and adapt to climate events in resource compromised areas.

Changing climate alters environmental, economic, and social factors that can, in turn, influence disparate patterns of premature mortality. Despite growing attention to the health equity and environmental justice implications of climate change, there has been less research examining the association between recurring climate-related events and population health. Drawing on a Social Determinants of Health (SDoH) approach emphasizing the links between socioeconomic and environmental factors in population health [[7], [8], [9]], we aimed to identify the differential associations of climate change with health outcomes. Specifically, we examined the relationship between multiple climate-related extreme events and age-adjusted years of potential life lost across two racial subgroups, non-Hispanic Blacks and non-Hispanic Whites (Black and White, hereafter), to discern difference between two populations living within the same sets of counties in the US.

In the US, racial and economic health disparities persist, and since 2014, life expectancy has been declining [10]. Geographic location largely shapes a person’s exposure to climate events and changing weather, and consequentially, the health risks from climate change are not uniformly distributed across race/ethnic and socioeconomic statuses. Chronic divestment in marginalized communities leaves infrastructure unprepared for climate-related extreme events and potentially places disadvantaged minority groups at a heightened risk for climate events. Additionally, climate events exacerbate existing inequality in access to resources, as these disasters have large socioeconomic impacts [11]. Counties serve as a particularly relevant unit of analysis; they are a key level of government tasked with responding to the types of natural hazards that climate change will exacerbate.

## 2 Materials and methods

To examine the relationship between climate extremes and population health outcomes, we focused on age-adjusted years of potential life lost (YPLL), a commonly used measure of premature mortality, derived from the County Health Rankings and Roadmaps (CHRR) project [12]. This measures the number of years of potential life lost for residents younger than age 75 per 100,000 people. It is a continuous, normally distributed variable. We analyze YPLL for two primary reasons. First, population-based climate-mortality data remain under-reported and unreliable, with large variations in estimates. Second, the health consequences from climate-related extremes are diverse and include both direct and indirect pathways to mortality; YPLL allows us to examine the association between premature mortality and climate extremes across diverse exposures to multiple climate events, without limiting the association to direct causes of death. The 2021 CHRR data provide aggregated mortality data by race/ethnicity from the 2017–2019 National Center for Health Statistics as drawn from the National Vital Statistics System raw data files. We focused on the three-year aggregated data to precede the SARS COVID-19 pandemic. We utilized two subgroup measures as dependent variables from the 2021 CHRR data release: (1) YPLL for Black residents and (2) YPLL White residents.

### 2.1 Measuring climate extremes

Our main independent variable is a novel measure of climate-related extreme events. Constructing local measures of climate change related events is complex; droughts, extreme temperatures, floods and other climate related disasters occur on a range of spatial and temporal scales. Climate and weather are not synonymous, however, climate change will amplify severe weather and intensify natural hazards, with established links between frequent and intense weather events and climate change over time [11,13].

While deviations from long-term (often 100-year) historical averages are typically used to identify climate extreme events [11,14], we used the last decade as a starting point to construct measures of climate extremes. Climate change continues to lead to more frequent and damaging extreme events [[15], [16], [17]]. For example, the Intergovernmental Panel on Climate Change warn of multiple and recurrent climate extremes and their interactions with other non-climate-related risks. Accordingly, we utilized climate data from the previous decade to examine appropriate lag times between climate extremes and subsequent premature mortality.

We developed three county-level climate measures using data from 2010 - 2019: *extreme heat, drought*, and *damaging storms*. Importantly, we aimed to develop an intuitive and simple measure that provides a useful understanding of complex data to multiple stakeholders (e.g., county planning committees). Data came from the Centers for Disease Control and Prevention’s (CDC) National Environmental Public Health Tracking Network (Tracking Network) [18]. This system contains data on environmental hazards, including data on extreme heat events. Heat events are defined as temperatures exceeding 100-degrees Fahrenheit for at least two consecutive days. We compiled data on the number of extreme heat events within each county for a calendar year to calculate the average heat events in each county from 2010–2019. This average measure allows the creation of a dichotomous county-level variable indicating above average heat events in this time-period.

We constructed a similar measure for drought based on the total number of weeks in which 50 % or more of a county was in severe, extreme, or exceptional drought as reported in the United States Drought Monitor (USDM) and accessed from the CDC’s Tracking Network. We calculated an average measure of county-level drought across the decade. Following, we developed a dichotomous indicator noting counties with above-average drought conditions.

Extreme storms was our third climate-related measure. Using the NOAA’s National Center for Environmental Information’s Storm Events database, a resource that documents severe and damaging storms, we calculated the average number of severe and damaging storms reported in a county per year, 2010–2019. This database includes cold-weather events, convective hazards (including precipitation and floods), marine-related events, and other hazards, such as wildfires. We removed any heat-related storm data from this measure to avoid redundancies. Again, we constructed a measure to identify which counties experienced above-average damaging storms.

Finally, the three binary variables of above-average extreme heat events, drought, and damaging storms were combined to create a summed overall indicator of *climate extremes* from 2010–2019 and categorized into 0, 1, 2, or 3 climate events.

### 2.2 Control variables

To isolate the independent contribution of climate exposures, we included county-level aggregate control variables based on the SDoH literature to account for sociodemographic characteristics, socioeconomic status (SES), health behaviors, and social capital differences. Several controls were derived from CHRR 2020 data, the year prior to the mortality data to ensure temporal ordering. Demographic controls included the percentage of *Black population* within a county and an index of dissimilarity (i.e., percentage of Black to White residents in a county) to measure *residential segregation*. SES control variables included measures of poverty, economic inequality, unemployment, and educational attainment, all established determinants of population health [19]. *Poverty* was measured as the percentage of people in a county below the 100 % federal poverty line estimate. *Income inequality* was the ratio of household income within a county at the 80th percentile to the income at the 20th percentile. *Unemployment* was measured as the percentage of adults currently unemployed in a county who are seeking work. *Educational attainment* measured the percentage of people age 25–44 in a county with at least some college education, including vocational/ technical schools, junior colleges, and four-year universities. Health controls accounted for behavioral and access issues and included the county-level percentage of *current smokers* and the percentage of individuals under age 65 without any *health insurance coverage*.

Additionally, we included *social capital*, another established determinant of population health, as a control variable [[20], [21], [22]]. This index was based on the per capita number of associations and membership groups in each county, voter turnout, and US Decennial Census participation [23]. Table 1 describes the variables and data sets used in this analysis.

**Table 1:** Variable description.

Variable	Description & Source
Black and White Premature Death – Years of Potential Life Lost	The number of years of potential life lost before the age of 75 per 100,000 residents, by racialized group. County Health Rankings and Roadmaps Project (CHRR) use mortality files from the National Center for Health Statistics (Mortality All County [micro data]) to calculate age adjusted premature mortality. We downloaded CHRR 2021 data release, which used microdata from 2017–2019 to construct measures of age adjusted premature mortality by two racialized group (non-Hispanic Blacks and non-Hispanic Whites).
Climate Extremes	A categorical measure noting if a county has experienced above-average heat events, drought, and damaging storms over the period of 2010–2019. This measure is constructed by identifying heat events per county per year from 2010–2019 and calculating the average heat events for counties in this time period. Next, we identify which counties have experienced above-average heat events in that time period. Similar steps were followed for drought and damaging storms and this allowed us to construct our categorical measure. Heat and drought data are drawn from the CDC’s National Environmental Public Health Tracking data. Storm data is drawn from the National Oceanic and Atmospheric Administration's Storm events database.
Sociodemographic	
Non-Hispanic Black	Percentage of population that is non-Hispanic Black or African American. Source: Census Population Estimates 2018. Downloaded from the CHRR 2020 data release.
Residential Segregation	Index of dissimilarity where higher values indicate greater residential segregation between Black and White county residents. Source: American Community Survey, 5-year estimates. 2014–2018. Downloaded from the CHRR 2020 data release.
Socioeconomic	
Poverty	Percentage of the people in a county below the federal poverty estimate. Federal poverty level varies depending on the size of a household. The Agency for Toxic Substances and Disease Registry (ATSDR) calculates this percent by checking poverty status in a county and dividing the number of people below federal level by the total population in county. They rely on American Community Survey data from 2010–2014 to construct this mean. Source: Centers for Disease Control and Prevention and the Agency for Toxic Substances and Disease Registry’s Social Vulnerability Index.
Income Inequality	Ratio of household income at the 80th percentile to income at the 20th percentile. Source: American Community Survey – 5 year estimates (2014–2018). Downloaded from the CHRR 2020 data release.
Unemployment	Percentage of unemployed population (16+) in the county who are looking for work. Bureau of Labor statistics (2018). Downloaded from the CHRR 2020 data release.
Educational Attainment	Percentage of adults age 25–44 with some college education. Source: American Community Survey, 5-year estimates. 2014–2018. Downloaded from the CHRR 2020 data release.
Health	
Smoking	Percentage of current smokers by county. Source: Behavioral Risk Factor Surveillance System 2014–2018. Downloaded from the CHRR 2020 data release.
Uninsured	The percentage of people in a county 65 and under without health insurance (reported by Small Area Health insurance estimated. Reported in 2017 (included in CHRR 2020 data release). Downloaded from the CHRR 2020 data release.
Social Capital	An index of four county factors, standardized to have a mean of zero and a standard deviation of 1: (1) Number of Associations per 1000 including religious, civic, social, business, political, professional, labor, bowling, fitness, golf, and sports (2) 2008 Voter turnout, (3) 2010 census response rate, (4) Number of nonprofit organizations not internationally focused per 10,000. Compiled by Rupasingha, Goetz and Freshwater (2006, with updates).

Our analysis capitalized on the availability of local level aggregated YPLL data by racial subgroup. Confidentiality concerns limited the number of counties included in this analysis, but health data by subgroup provided an opportunity to examine the health equity consequences of recurrent climate extreme events. Analyzing the subgroup differences provided a clearer focus on health equity, a critical aspect needed in any climate or health policy. We restricted our analysis to the same set of counties reporting premature mortality for both Black and White subgroups, resulting in an analytical sample of 1,358.

First, we presented summary statistics of the variables for all counties included in this analysis. Second, we presented means across the climate extremes. Third, we used multivariable regression analysis to model the cross-sectional relationship between climate extremes and years of life lost prematurely across two racial subgroups, controlling for county-level sociodemographic, socioeconomic, health, and social capital variables. The formal model specification is below where Y*i* is YPPL by racial/ethnic subgroup, β*_j_* CE*_ji_* notes the categories of climate extreme events and X*_ki_* is the vector of control variables guided by the SDoH literature.

$$Y_{i}=\;\beta_{0}+\;\sum_{j=1}^{3}\beta_{j}CE_{ji}+\;\sum_{k=1}^{K}\gamma_{k}\;X_{ki}+\;\varepsilon_{i}$$

All analyses were conducted in Stata SE 18. Maps were created using ESRI ArcMap Desktop 12.0.

## 3 Results

**Fig. 1:**
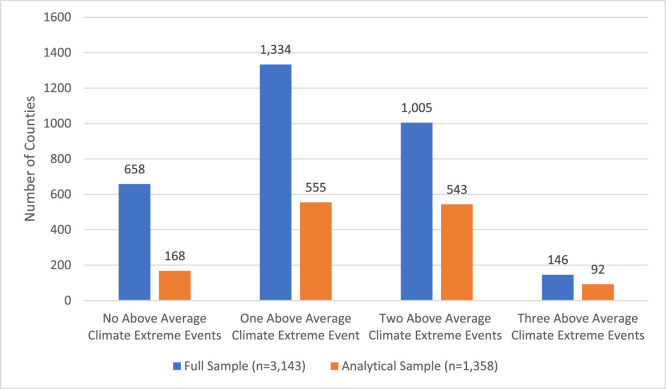
Frequency of above average climate extreme events (2010 −2019).

^Note: Full sample includes all counties in the United States. Analytic sample includes the counties where health data is reported by racial subgroup (1358 counties)^

**Fig. 2:**
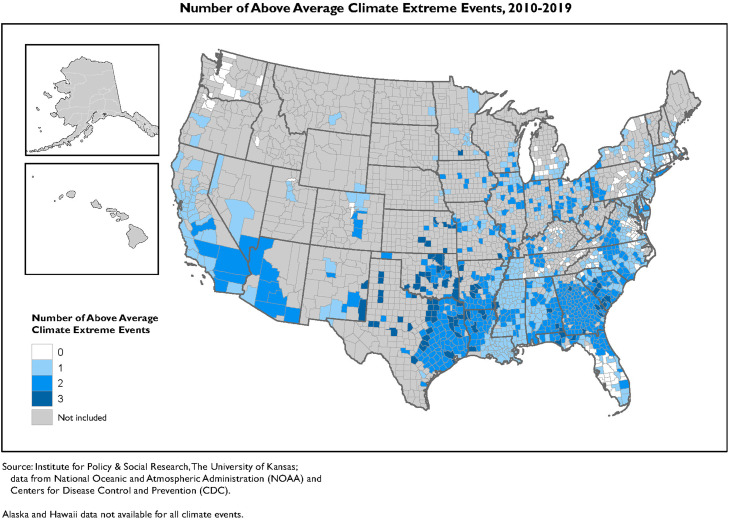
Above average climate extreme events, 2010–2019.

Fig. 1 presents the distribution of counties experiencing above average climate extremes. We display the frequency of climate extremes for all counties in the US, the full sample, and for the subset of counties in our analytic sample (the counties where health outcome data by subgroup is available). Across the US, 658 counties experienced no above-average climate extremes, 1,334 counties experienced one above-average event, 1,005 counties experienced two above-average events, and 146 counties experienced above-average heat events, drought, and damaging storms. Fig. 2 shows the geographic patterns of above average heat, drought and damaging storms for the counties in our analytical sample. While risks of drought, extreme heat, precipitation fluctuations, storms, and flooding have distinct regional patterns, we highlight areas that have experienced multiple extreme climate events. There were clear patterns of accumulated climate risk in the Southwest and Southeast. Additionally, the Mississippi Delta region, an area characterized by high levels of socioeconomic and health disadvantages [24], had several counties experiencing multiple recurrent extreme climate events.

Table 2 presents descriptive statistics for the variables. Table 3 provides mean values for all variables by climate extremes. One-way analysis of variance tests indicated statistically significant differences in average values across categories of climate extremes for each of the variables. Table 4 reports the results of our analysis of YPLL regressed on climate extremes by racial subgroup, controlling for sociodemographic, SES, health, and social capital variables. The models for Black and White YPLL analyzed the same counties, however, with divergent results. Above-average climate extremes were linearly associated with statistically significant years of Black premature mortality at each level of climate events. The change from 1 to 2 climate events does not show a statistically significant difference for Blacks, albeit both remain statistically significant from no events. Yet, when moving to 3 climate events, YPLL relative to no events (reference) increased by approximately 37 % (*p* ≤ 0.001) and 52 % (not statistically significant) for Blacks and Whites, respectively. Within the same counties, climate extremes were associated with an elevated risk of premature mortality for Black populations, but this risk was not evident for White populations.

**Table 2:** Descriptive statistics.

	Average	Standard Deviation	Minimum Value	Maximum Value
Health Outcomes				
Black YPLL	11,460.38	3558.13	3046.90	32,122.24
White YPLL	8611.55	2438.42	2389.77	28,201.23
Climate Extremes (overall)	1.41	0.79	0.00	3.00
1 (above average event)	0.41	0.49	0.00	1.00
2 (above average events)	0.40	0.49	0.00	1.00
3 (above average events)	0.07	0.25	0.00	1.00
Sociodemographic				
Non-Hispanic Black	18.53	17.02	0.59	85.41
Residential Segregation	41.31	14.98	0.04	83.03
Socioeconomic				
Poverty	18.20	6.68	3.80	46.60
Income Inequality	4.76	0.77	3.08	11.97
Unemployment	5.99	2.30	1.80	23.40
Educational Attainment	57.93	12.05	20.85	90.34
Health				
Smoking	17.90	3.42	5.91	31.85
Percent Uninsured	11.58	4.68	2.26	26.85
Social Capital	−0.53	0.88	−3.93	17.44
N	1358

**Table 3:** Average variables overall and by categories of climate extreme events.

		Number of Above Average Climate Extreme Events			
	Overall	0	1	2	3
Health Outcomes					
Black YPLL^1***^	11,460.38	9987.96	11,509.71	11,774.38	12,050.45
White YPPL^⁎⁎⁎^	8611.55	7895.55	8496.61	8936.05	8705.05
Sociodemographic					
Non-Hispanic Black^⁎⁎⁎^	18.53	11.7	19.17	20.53	15.57
Segregation^⁎⁎^	41.31	44.03	42.36	39.20	42.34
Socioeconomic					
Poverty^⁎⁎⁎^	18.20	15.35	18.04	19.26	18.15
Income Inequality^⁎⁎⁎^	4.76	4.49	4.78	4.83	4.63
Unemployment^⁎⁎^	5.99	5.40	6.21	5.99	5.64
Educational Attainment^⁎⁎⁎^	57.93	60.59	58.94	55.92	58.70
Health					
Smoking^⁎⁎⁎^	17.90	16.90	17.91	18.27	17.50
Uninsured ^⁎⁎⁎^	11.58	10.07	10.19	12.95	14.72
Social Capital^⁎⁎⁎^	−0.53	−0.37	−0.477	−0.60	−0.79
N = 1358					

1 YPPL: Years of Potential Life Lost before age 75.

Significance tests (F-statistics) from one way analysis of variance tests highlighting between group differences in means across climate extremes (* p < 0.05, ** p < 0.01, *** p < 0.001).

**Table 4:** Multivariable regression analysis of race/ethnic years of potential life lost (YPLL) on Climate Extremes.

	Model 1 Non-Hispanic Black YPLL		Model 2 Non-Hispanic White YPLL	

Climate Extremes				
1	1032.39	(233.59)⁎⁎⁎	129.62	(175.50)
2	1026.33	(258.30)⁎⁎⁎	146.73	(176.44)
3	1629.81	(352.76)⁎⁎⁎	269.77	(210.25)
Sociodemographic				
Non-Hispanic Black	−28.27	(8.07)⁎⁎⁎	−3.31	(5.26)
Residential Segregation	30.03	(6.09)⁎⁎⁎	−5.17	(3.68)
Socioeconomic				
Poverty	100.41	(24.65)⁎⁎⁎	5.65	(14.44)
Income Inequality	254.00	(129.70)+	−152.09	(90.78)+
Unemployed	120.88	(63.62)+	116.53	(40.67)⁎⁎
Educational Attainment	−32.29	(11.34)⁎⁎	−67.65	(6.39)⁎⁎⁎
Health				
Smoking	310.53	(39.82)⁎⁎⁎	280.87	(19.40)⁎⁎⁎
Uninsured	61.90	(25.93)*	43.63	(13.27)⁎⁎
Social Capital	750.76	(314.05)*	−0.94	(91.44)
				
Constant	2046.66	(1380.77)	7063.41	(790.89)⁎⁎⁎
N	1358		1358	
R^2^	0.332		0.551	
adj. R^2^	0.326		0.547	

Standard errors in parentheses.

+ p < 0.10.

⁎ p < 0.05.

⁎⁎ p < 0.01.

⁎⁎⁎ p < 0.001.

Control variables were consistent with a SDoH framework. However, there were a few inconsistencies across race/ethnic groups. For Black populations, higher rates of poverty were related to more years of life lost in aggregate. This was, however, not a statistically significant relationship associated with White premature mortality. Income inequality also operated differently for Black and White health outcomes. Higher inequality was linked to higher premature mortality for Black health, but lower levels of income inequality were linked to higher White premature mortality. Income levels for White households were disproportionately in the top quartiles of income, while income levels for Black households were disproportionately in the bottom 10 % of income distributions [25]. Areas with high income inequality are likely to be counties with racial disparities in income. This could result in a health-protective effect of income inequality on White health. Controls for education, unemployment, smoking, and health care access were consistent with past research on the SDoH. Higher levels of social capital in a county were associated with higher levels of premature mortality. However, social capital was only statistically significant for Black premature mortality, and the direction of the relationship contrasts with previous findings in the literature [26,27].

## 4 Discussion and conclusion

We merged an array of county-level data to better understand how collectively, extreme heat, severe drought, and damaging storms were associated with racial patterns of population health. Using race-specific mortality outcomes, we examined the health equity impacts of a novel measure of recurrent climate-related events on age-adjusted-premature mortality and found that county-level climate-related extremes were associated with increased Years of Potential Life Lost but were only statistically significant for Black populations. Importantly, these associations persist even controlling for other SDoH, suggesting climate exposures should be considered independent additional contributors to health. Similar to other research, we find that community-level conditions may have unique associations for race/ethnic mortality outcomes [28], suggesting a need for climate models that focus on specific community needs.

Our results indicate the importance of examining climate change at the county level. Local-level data is critical to assessing health risks and tailoring effective interventions, but many local governments lack the capacity to gather and analyze this data [29,30]. To meet specific community needs, leaders and decision makers in states and localities need more, easy to understand, local information on the converging health threats from climate changes [2,31]. In the US, county governments authorize billions of dollars of spending each year, including investments in community resilience, mitigation, and adaptation in the face of increasing climate risks [32]. Crafting policies that promote health and equity requires understanding the interconnections between increased climate risks, racial disparities, and health outcomes.

Our research provides evidence that could improve health equity in the face of recurrent climate-related events, highlighting differential patterns when comparing the determinants of Black and White premature mortality. This result is not surprising considering communities of color have been identified as highly vulnerable to climate change’s health effects [4,5]. In part, net of other predictors of health, we find that White mortality is not statistically related to recurrent climate extreme events, highlighting potential advantages to mitigate climate risk for those with more socioeconomic resources. This may also be due to a host of unobserved factors such as community preparedness or resilience, information networks, residential selection issues, social networks, or climate event related mobility.

Previous work emphasizing disparities in climate change’s health effects at the local level focuses on limited communities and singular types of climate exposures [33]. Our results are relevant to a broader set of communities and quantify the effect of multiple overlapping climate events, particularly for Black premature mortality.

However, some caveats are important to note. First, our measure of climate extreme events underestimates climate impacts and local experiences with climate extremes and provides a conservative measure of climate extremes over a decade. We construct measures to note if a county has experienced above-average heat, drought or damaging storms over a 10-year period (2010–2019). Future research would benefit from investigation of additional years of events. Nonetheless, while this approach may include a loss of detailed information about the specific nature of climate-related events, we present a novel, straightforward approach that is theoretically relevant for understanding how multiple exposures can be associated with deleterious health outcomes. Additional testing using alternative operationalizations, including higher thresholds for exposure to climate events (75th percentile) and standardized climate indices, produced similar results, demonstrating robustness of our findings. Although we controlled for the most critical SDoH, unmeasured, unobserved factors may still confound results.

Second, our analysis is limited to the counties reporting both Black and White premature mortality resulting in a loss of observations. Remaining counties in our subgroup analysis have enough residents to aggregate mortality data and maintain privacy. In other words, these tend to be more densely populated counties within urban areas, reducing generalizability too more rural counties. Nonetheless, the counties that remain for the subgroup analysis highlight existing racial health disparities. While we note this caution, we contend that it is imperative to use county-level data to develop a better understanding of climate events and health outcomes at a local level. Beyond this, an important component of that understanding should be rooted in the SDoH and consideration of existing racial and economic health disparities. Subgroup analysis –even when sample sizes are reduced – provides needed evidence to state and local decision makers. Climate change has socioeconomic impacts that widen existing disparities [11], making it critical to address these compounded health risks.

Our research provides foundational evidence on the local-level health impacts of climate change. Climate change and health research should integrate overlapping risks from climate exposure and subgroup analysis. Future research could build upon our findings in several ways. Similar analyses could examine how these factors impact other important health outcomes, especially mental health measures or cause-specific factors. Further research should explore the mixed results for social capital. Our finding contrasts with literature heralding social capital as a tool to build community resilience in the face of climate change[34], demonstrating social connections may exacerbate climate change’s damages to marginalized populations. Even though the included variables are temporally assigned, in the case of Black communities with historically high levels of premature mortality, it is possible that social capital measures of social associations include programs targeted at improving health, suggesting potential reverse causality.

Our results have policy implications and emphasize the need for local governments to attend to the impacts of climate change on vulnerable race/ethnic groups. Interventions are needed to mitigate the harms created by policies that have historically marginalized and segregated communities of color which are likely compounded through repeated exposure to climate change [35]. Local decision makers could advance environmental justice through interventions to increase health care access and monitor the health equity outcomes of these programs in the face of increasing climate risks. Community-based solutions to climate-related health problems should empower and engage community members in decision-making [[36], [37], [38], [39]]. Leveraging data and technical expertise to partner with communities could reduce health disparities arising from climate change [40]. Without addressing the compounded risks some face, climate change will continue to deteriorate health, wealth, and equity. Providing local decision makers evidence on the correlation between climate events, protective resources, and health outcomes is critical to mitigating the detrimental health effects of climate change and promoting health equity.

## CRediT authorship contribution statement

**Dorothy M. Daley:** Writing – review & editing, Writing – original draft, Supervision, Project administration, Methodology, Investigation, Funding acquisition, Formal analysis, Data curation, Conceptualization. **Margaret H. Swenson:** Writing – review & editing, Writing – original draft, Validation, Methodology, Investigation, Formal analysis, Data curation. **Jarron M. Saint Onge:** Writing – review & editing, Writing – original draft, Methodology, Investigation, Funding acquisition, Formal analysis, Data curation, Conceptualization.

## Declaration of competing interest

The authors declare that they have no known competing financial interests or personal relationships that could have appeared to influence the work reported in this paper.
